# Commentary on: The use of burosumab to treat autosomal-recessive hypophosphatemic rickets type 2: rationale and a first clinical experience

**DOI:** 10.1007/s40620-024-02057-9

**Published:** 2024-08-20

**Authors:** Frank Rutsch, Isidro B. Salusky, Carlos R. Ferreira

**Affiliations:** 1 Department of General Pediatrics and Center for Rare Diseases, Muenster University Children’s Hospital, Muenster, Germany; 2 Department of Pediatrics, David Geffen School of Medicine at UCLA, Los Angeles, CA USA; 3 Metabolic Medicine Branch, National Human Genome Research Institute, National Institutes of Health, Bethesda, MD USA

We read the article by Parolin et al. in the February issue of this journal with great interest. In their paper, the authors describe the use of burosumab in a 4-year-old girl with autosomal recessive hypophosphatemic rickets type 2 (ARHR2) [1]. The authors discuss the rationale of burosumab treatment in their case thoughtfully and mention the fact that generalized arterial calcification of infancy (GACI) and autosomal recessive hypophosphatemic rickets type 2 share the same genetic changes in *ENPP1*, while generalized arterial calcification of infancy is associated with calcifications and intima proliferation in large and medium-sized vessels, as well as periarticular calcifications [2]. They also mention that patients with generalized arterial calcification of infancy frequently develop autosomal recessive hypophosphatemic rickets type 2 during adolescence and that the administration of burosumab could potentially be harmful by reducing FGF23 levels, which might worsen arterial calcifications. Indeed, we are aware of such complications, seen in a boy initially diagnosed with generalized arterial calcification of infancy, who, after developing hypophosphatemic rickets, was treated with burosumab and developed new cardiac calcifications during treatment [3].

The question arises, if there is a "pure" form of autosomal recessive hypophosphatemic rickets type 2 without any signs of arterial calcification or cardiovascular involvement, which would not pose the risk of the potential side effects of blocking FGF23 activity. In our experience, which includes ENPP1-deficient patients who participated in the recently published Natural History Study [2], there are certainly a few patients who were considered as “pure” autosomal recessive hypophosphatemic rickets type 2, who, when evaluated in more detail, had signs of cardiovascular involvement. Most commonly, these patients had some kind of valve involvement (a murmur that does not disappear over time, valvular stenosis, etc.). In some cases it might be chronic hypertension, eventually diagnosed as likely related to renal vascular stenosis; or it may be left ventricular hypertrophy, or arterial calcification, which was not detected initially. Thus, we believe that in many cases, “pure” autosomal recessive hypophosphatemic rickets type 2 is not so pure after all. Furthermore, the current imaging techniques utilized in clinical practice may fail to detect early vascular calcifications in children.

Therefore, the final statement of the authors: 'Albeit with the limitations of a case report, this paper describes the possibility of using burosumab effectively and safely even in patients with FGF23-dependent hereditary hypophosphatemic rickets other than X-linked hypophosphatemia, broadening the benefits of this therapy also to patients with different mutations' should be taken cautiously because, in our experience, there is no such thing as autosomal recessive hypophosphatemic rickets type 2 disease by itself.

Although burosumab might address renal phosphate wasting, it does not address the underlying causal mechanism of ENPP1-deficiency-related disease, which is a deficiency of extracellular inorganic pyrophosphate (PPi) and adenosine [4, 5], and which is not corrected with burosumab. Fortunately, a specific ENPP1 enzyme replacement therapy is emerging, the effectiveness of which must be proven in the clinical trials that are already underway [6].
